# Assessment of PEG-Asparaginase and Erwinia Asparaginase Activity Under Variable Storage Conditions

**DOI:** 10.3390/pharmaceutics18050618

**Published:** 2026-05-18

**Authors:** Søren Aleksander Friederici Dahl, Elke Hoffmann-Lücke, Birgitte Klug Albertsen, Eva Greibe

**Affiliations:** 1 Department of Emergency Medicine, Randers Regional Hospital, 8930 Randers, Denmark; 2 Department of Clinical Biochemistry, Aarhus University Hospital, 8200 Aarhus, Denmark; elkehoff@rm.dk (E.H.-L.); evagreib@rm.dk (E.G.); 3 Department of Clinical Medicine, Aarhus University, 8200 Aarhus, Denmark; biralber@rm.dk; 4 Department of Paediatrics and Adolescent Medicine, Aarhus University Hospital, 8200 Aarhus, Denmark

**Keywords:** PEG-Asparaginase, Erwinia asparaginase, pre-analytical stability, freeze–thaw cycles, whole blood, therapeutic drug monitoring, ALL, LBL

## Abstract

**Background**: PEG-Asparaginase and Erwinia asparaginase are enzyme-based anticancer therapies used in the treatment of acute lymphoblastic leukaemia (ALL) and lymphoblastic lymphoma (LBL), where adequate plasma enzyme activity is required for therapeutic efficacy. In many study groups, therapeutic drug monitoring is routinely applied due to pharmacokinetic variability and the risk of hypersensitivity reactions followed by increased clearance and insufficient treatment. In clinical practice, samples may be exposed to prolonged transport and variable pre-analytical conditions. Knowledge on pre-analytical stability is important for correct interpretation of PEG-Asparaginase and Erwinia asparaginase activity in plasma. This study aimed to evaluate the in vitro stability of PEG-Asparaginase and Erwinia asparaginase under pre-analytical conditions. **Methods**: Three experimental stability studies were conducted at two activity levels. Enzyme stability in plasma was assessed during storage at 4 °C and 20 °C for up to 14 days and following three freeze–thaw cycles. Stability in whole blood prior to centrifugation was evaluated over 24 h. Enzyme activity was measured using a validated spectrophotometric assay, and stability was defined as a deviation within ±15% of baseline activity. **Results**: Both enzymes remained stable in plasma for up to 14 days at 4 °C and 20 °C, and no clinically relevant reduction in enzyme activity of freeze–thaw cycling was observed. In whole blood, Erwinia asparaginase and high-activity PEG-Asparaginase remained stable for 24 h at 20 °C, whereas low-activity PEG-Asparaginase showed a reduction in activity of approximately 22%, mainly within the first two hours. **Conclusions**: PEG-Asparaginase and Erwinia asparaginase are stable in plasma for up to 14 days at room temperature, enabling shipment of plasma samples by mail. However, prompt centrifugation is recommended for samples with low PEG-Asparaginase activity to ensure accurate therapeutic drug monitoring.

## 1. Introduction

Acute lymphoblastic leukaemia (ALL) is the most common malignancy in childhood. Asparaginase-based therapy is an integral component of modern ALL treatment protocols, including regimens for relapsed and infant patients [1]. PEG-Asparaginase and Erwinia asparaginase exert their antileukemic effect through enzymatic depletion of circulating asparagine, an amino acid essential for the survival of leukemic lymphoblasts [2]. Sustained asparagine depletion requires adequate circulating enzyme activity; consequently, plasma asparaginase activity serves as a direct surrogate marker of systemic drug exposure [3].

Considerable interindividual pharmacokinetic variability, often driven by immunologically mediated reactions, may result in insufficient systemic exposure to asparaginase during therapy [4,5]. These immune-mediated events include three distinct phenomena: true hypersensitivity reactions with enzymatic inactivation, silent inactivation caused by neutralizing antibodies without clinical symptoms, and allergy-like reactions that mimic clinical hypersensitivity but occur without loss of enzymatic activity. Whereas true hypersensitivity and silent inactivation requires switching to an alternative asparaginase preparation, patients with allergy-like reactions may continue treatment with the same formulation provided that symptoms are mild [6].

Because these reactions cannot be reliably distinguished on clinical grounds alone, therapeutic drug monitoring (TDM) of plasma asparaginase activity is required to guide treatment decisions [4,7].

Accurate pharmacokinetic interpretation depends on reliable analytical measurements. Pre-analytical factors such as delayed centrifugation, prolonged transport at ambient temperature, or repeated freeze–thaw cycles may introduce degradation or variability in measured enzyme activity. These alterations may lead to misclassification of therapeutic exposure, erroneous estimation of drug clearance or inappropriate switching between enzyme preparations. Validation of pre-analytical stability is therefore essential for meaningful pharmacokinetic exposure assessment.

In Denmark, both domestic and international samples for TDM were analyzed at Aarhus University Hospital (AUH) and transported by postal service at room temperature for several days prior to measurement. Previous studies have demonstrated that asparaginase preparations are stable in human serum under various storage conditions, including several days at room temperature and prolonged storage at −20 °C and −80 °C [8,9]. Limited data from a small in vitro study further suggest that PEG-Asparaginase may remain stable in whole blood under short-term storage [10]. However, despite these findings, data on stability in EDTA plasma and whole blood under real-world pre-analytical conditions remain limited. The present study aimed to evaluate the in vitro stability of PEG-Asparaginase and Erwinia asparaginase at two activity levels under conditions reflecting routine clinical practice, including prolonged storage at 4 °C and 20 °C, repeated freeze–thaw cycles, and delayed centrifugation in whole blood. This evaluation is intended to support reliable TDM and accurate pharmacokinetic exposure assessment.

## 2. Materials and Methods

The study was performed at the accredited hospital laboratory at the Department of Clinical Biochemistry at AUH, Denmark (DS/EN ISO 15189 [11]) from October 2024 to May 2025.

### 2.1. Study Design

Three experimental studies were conducted to evaluate the in vitro stability of PEG-Asparaginase and Erwinia asparaginase in human EDTA plasma and whole blood under different pre-analytical conditions relevant to clinical practice. All experiments were conducted within two target activity ranges, low level (LL) (PEG-ASNase: 100–170 U/L; Erwinia ASNase: 70–130 U/L) and high level (HL) (PEG-ASNase: 610–990 U/L; Erwinia ASNase: 380–720 U/L), for both enzymes.

#### 2.1.1. Study 1—Temperature Stability Assessment

**Figure 1:**
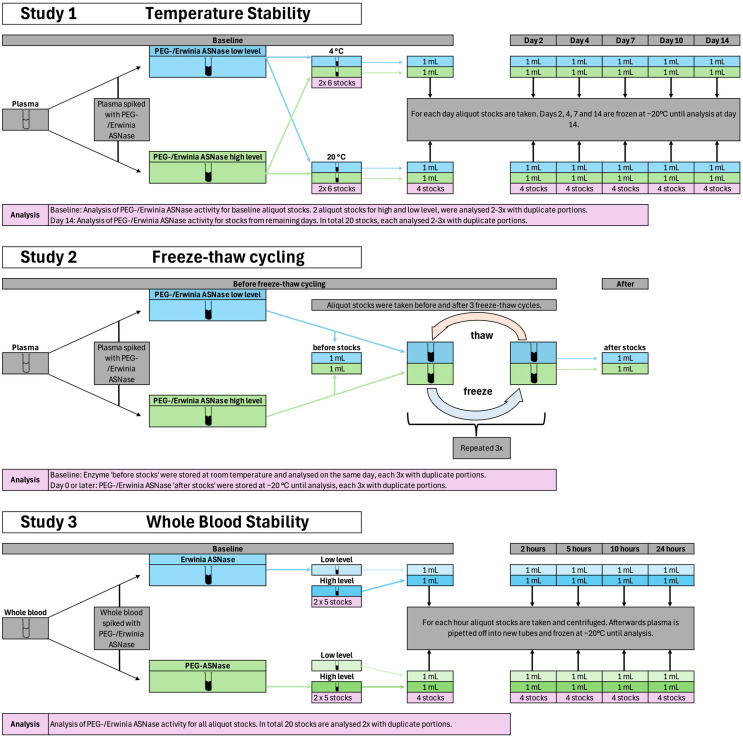
Schematic overview of the experimental study designs.

To investigate the effects of temperature on the stability of PEG-Asparaginase and Erwinia asparaginase in plasma, spiked stock solutions were prepared and stored at 4 °C and 20 °C for 14 days (Figure 1 (Study 1)). At day 0, 2, 4, 7, 10, and 14, aliquots were removed and stored at −20 °C to halt further enzymatic alteration.

Aliquots were analyzed twice at low level and three times at high level, yielding 10 independent measurements per time point. In total, 60 PEG-Asparaginase and 60 Erwinia asparaginase samples were included. Each sample was analyzed in duplicates.

#### 2.1.2. Study 2—Freeze–Thaw Cycling

To investigate the effects of repeated freeze–thaw cycles on enzyme stability, a freeze–thaw experiment was performed using spiked stock solutions for both PEG-Asparaginase and Erwinia asparaginase in plasma (Figure 1 (Study 2)). Prior to initiating the cycles, aliquots were obtained from each stock to serve as baseline controls. Baseline aliquots were analyzed on the day of preparation to minimize pre-analytical alteration. Baseline aliquots were stored at room temperature and analyzed within 1 h in triplicate. The remaining samples were subjected to three freeze–thaw cycles, each consisting of freezing at −20 °C followed by thawing at 20 °C. After the final cycle, samples were stored at −20 °C until analysis.

#### 2.1.3. Study 3—Whole Blood Stability Assessment 

To investigate the stability of PEG-Asparaginase and Erwinia asparaginase in whole blood, samples were spiked with either enzyme (Figure 1 (Study 3)). At baseline, two aliquots were prepared per time point and activity level, resulting in four aliquots per enzyme and 40 aliquots in total. Baseline samples were centrifuged at 2500 rpm for 5 min, after which plasma was collected and stored at −20 °C to prevent further enzymatic alteration.

After 2, 5, 10, and 24 h, samples were centrifuged under the same conditions, and plasma was collected and stored at −20 °C until analysis. Stability over the 24-h period was subsequently assessed.

### 2.2. Samples

PEG-Asparaginase (Oncaspar, 700 U/mL, Servier, Suresnes, France, CAS no.: 130167-69-0) and Erwinia asparaginase (Erwinase, 4000 U/L, Proton Biopharma Limited, Salisbury, UK, CAS no.: 9015-68-3) were purchased from the hospital pharmacy as part of the routine clinical practice at the Department of Clinical Biochemistry, AUH. Lower- and higher-level stock solutions were prepared by serial dilution in whole blood and EDTA plasma, as described in the study design. The lower level was chosen for its clinical relevance (therapeutic target: 100 U/L), whereas the higher level was selected to assess the effect of increased activity on stability.

Blood used in the experiments was obtained from a healthy donor.

### 2.3. Assays

Enzyme activity was quantified by measuring substrate formation at the reaction’s maximum rate using the indooxine method [8], with absorbance measured on a spectrophotometer (BioTek Elx808 Absorbance Microplate Reader, BioTek Instrument (AH Diagnostics), Winooski, VT, USA).

Samples were analyzed undiluted and in duplicate. The analytical measuring range for both PEG-Asparaginase and Erwinia asparaginase was 55–1045 U/L.

All analyses were performed by trained technicians as part of accredited routine procedures at the Department of Clinical Biochemistry, AUH. The limit of detection (LoD) and limit of quantification (LoQ) for both enzymes were 0.5 U/L and 10 U/L, respectively. Analytical precision, evaluated using quality control materials, ranged from 5–7%, based on 130 PEG-Asparaginase runs and 50 Erwinia asparaginase runs. The laboratory participates in an external quality assurance program (Stiftung für Pathobiochemie und Molekulare Diagnostik, Referenzinstitut für Bioanalytik) with satisfactory performance. Both assays were linear up to 1000 U/L.

### 2.4. Ethical Approval

According to the Danish Health Care Act, the study was exempted from approval by regional health ethics committees and the General Data Protection Regulation, as only anonymized blood from healthy donor was used.

### 2.5. Statistical Analysis

Statistical and graphical analyses were performed using R (version 4.5.1) within Rstudio (version 2025.09.2+418). Due to the small number of observations, normality assumptions were not met. Median activities were calculated for all time points. Percentage differences (%PD) were calculated using the mean baseline activity as reference. For comparison of data across more than two time points, the Friedman test (non-parametric equivalent of repeated measures ANOVA) was used to assess changes over time within each stock. Where *p* < 0.05, post hoc analysis was performed using the Wilcoxon signed-rank test. To control the family-wise error rate, *p*-values were adjusted using the Holm–Bonferroni method. For comparisons between two time points, the Wilcoxon signed-rank test was applied.

Measurements outside the analytical range (55–1045 U/L) and duplicate measurements with a coefficient of variation (%CV) >20% were excluded. One additional value was excluded due to a pre-analytical pipetting error. To retain data, missing values were imputed using median activities when ≤2 observations were missing at a given time point.

### 2.6. Acceptability Criteria

As no generally accepted maximal permissible difference (MPD) has been established, a threshold of 15% was applied to account for combined pre-analytical and analytical variability. Percentage differences exceeding this threshold were interpreted as clinically relevant instability.

## 3. Results

This study evaluated the stability of PEG-Asparaginase and Erwinia asparaginase under various storage conditions. The results of the three studies are presented below.

### 3.1. Study 1

**Figure 2:**
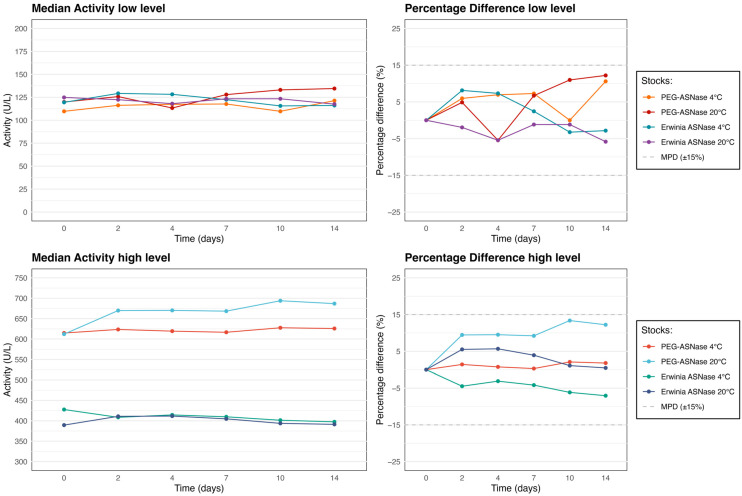
Median enzyme activity (U/L) and percentage difference from baseline (%PD) over 14 days for two activity ranges of PEG-Asparaginase (PEG-ASNase) and Erwinia asparaginase (Erwinia ASNase) stored at 4 °C and 20 °C in plasma (Study 1).

Results are summarized in Table 1 and Figure 2. Both PEG-Asparaginase and Erwinia asparaginase showed statistically significant changes under selected conditions, for example at low concentrations at 4 °C (Erwinia asparaginase) and at 20 °C (PEG-Asparaginase). However, both enzymes remained stable in plasma for up to 14 days at 4 °C and 20 °C, with percentage differences below 15%, i.e., within the predefined MPD.

### 3.2. Freeze–Thaw Stability

**Figure 3:**
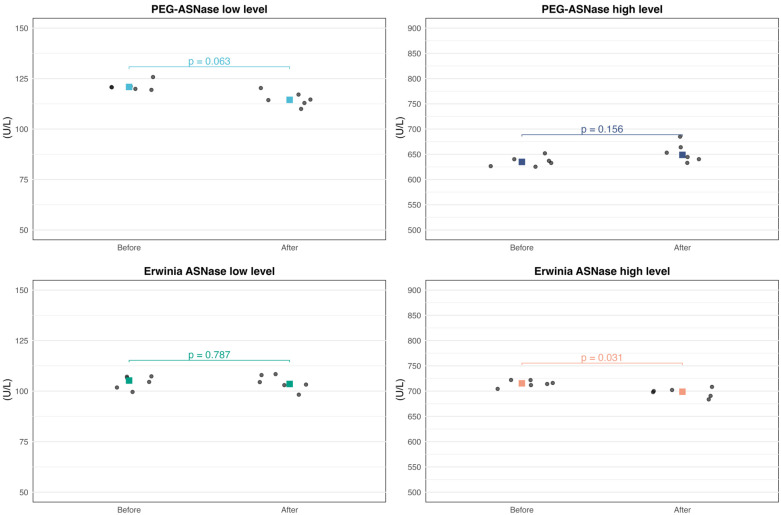
Enzyme activity (U/L) before and after three freeze–thaw cycles for PEG-Asparaginase and Erwinia asparaginase at two activity ranges (Study 2). Individual observations are shown in grey, and median activities are indicated in color. *p*-values were calculated using the Wilcoxon signed-rank test.

Results from the freeze–thaw study are summarized in Table 2 and Figure 3. Both PEG-Asparaginase and Erwinia asparaginase remained stable after three freeze–thaw cycles, with PD% within the 15% MPD.

### 3.3. Whole Blood Stability

**Figure 4:**
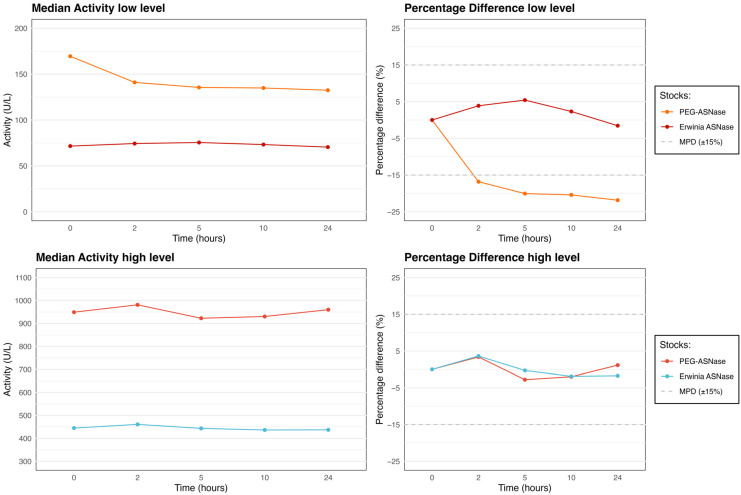
Median enzyme activity (U/L) and percentage difference from baseline (%PD) over 24 h for PEG-Asparaginase and Erwinia asparaginase at two activity ranges in whole blood stored at 20 °C (Study 3).

Results from the 24-h whole blood study are summarized in Table 3 and Figure 4. Low-level PEG-Asparaginase showed a marked decrease from 169.5 U/L at baseline to 132.5 U/L at 24 h (−21.8%) (*p* = 0.016). The largest decline (%PD: 16.8%) occurred between baseline and 2 h.

High-level PEG-Asparaginase and Erwinia asparaginase remained stable throughout the 24-h period, with PD% not exceeding the MPD.

## 4. Discussion

In this study, we evaluated the pre-analytical stability of PEG-Asparaginase and Erwinia asparaginase under conditions relevant for TDM. Both enzymes remained stable in EDTA plasma during storage at 4 °C and 20 °C for up to 14 days, with changes below 15%, and after three freeze–thaw cycles. Although statistically significant changes were observed at selected time points, none exceed the predefined threshold for clinically relevant change. In contrast, low-activity PEG-Asparaginase in whole blood showed a decline in enzyme activity prior to centrifugation, decreasing by 16.8% within the first two hours and exceeding 20% after 24 h.

The observed plasma stability across temperature conditions supports the robustness of enzyme activity measurements despite prolonged transport. This is clinically relevant in centralized laboratory settings, where samples may undergo several days of transport prior to processing. Similarly, the absence of clinically relevant changes after freeze–thaw cycling indicates that routine laboratory handling is unlikely to compromise measurement reliability, including for stored research samples. Although non-parametric testing identified significant differences at some time points, these did not persist after adjustment. Importantly, all observed variations remained within the predefined ±15% stability threshold.

The observed decline in low-level PEG-Asparaginase in whole blood may have clinical implications. Activities around 100 U/L represent therapeutic cut-offs for exposure classification, and even moderate reductions may lead to misclassification as subtherapeutic exposure [12]. Underestimation of enzyme activity due to pre-analytical degradation may therefore prompt unnecessary treatment modifications and distort assessment of systemic drug exposure. These findings underscore the importance of prompt centrifugation, ideally within two hours. A previous study did not demonstrate a loss of PEG-Asparaginase activity under comparable storage conditions, although this was assessed at higher activity levels (300 and 11,250 U/L) [10]. Consistent with these findings, our data also indicate stability at higher PEG-asparaginase concentrations, suggesting that clinically relevant loss of activity is primarily restricted to lower levels.

Strengths of the study include its clinically relevant design, evaluation of therapeutic activity levels, and use of an accredited assay with well-characterized analytical precision. By reflecting real-world transport and handling conditions, the findings provide practical support for reliable TDM implementation.

Limitations include the use of spiked samples rather than patient-derived material and the relatively small sample size. Future studies should include patient-derived samples to further validate these findings.

In conclusion, PEG-Asparaginase and Erwinia asparaginase are stable under most pre-analytical conditions relevant to clinical practice. Plasma-based TDM appears robust despite prolonged storage and freeze–thaw handling. However, reduced activity at low PEG-Asparaginase levels in whole blood highlights the importance of timely centrifugation to avoid underestimation of systemic exposure.

## Figures and Tables

**Table 1:** Median enzyme activity (U/L) over time for PEG-Asparaginase and Erwinia asparaginase stocks stored at 4 °C and 20 °C in plasma. Percentage difference from baseline is shown in parentheses (%PD). *p*-values were calculated using the Friedman test.

		Baseline (U/L)	Day 2 (U/L)	Day 4 (U/L)	Day 7 (U/L)	Day 10 (U/L)	Day 14 (U/L)	*p*-Values
PEG-ASNase	Low level 4 °C	109.7	116.3 (6.0%)	117.4 (6.9%)	117.7 (7.3%)	109.7 (0.00%)	121.4 (10.6%)	0.326
	Low level 20 °C	119.9	125.8 (4.9%)	113.4 (−5.5%)	128.0 (6.7%)	133.0 (11.0%)	134.6 (12.2%)	0.002
	High level 4 °C	614.8	623.5 (1.4%)	619.4 (0.7%)	616.6 (0.3%)	627.6 (2.1%)	625.8 (1.8%)	0.095
	High level 20 °C	612.0	669.8 (9.5%)	670.3 (9.5%)	668.4 (9.2%)	693.8 (13.4%)	686.8 (12.2%)	0.003
Erwinia ASNase	Low level 4 °C	119.5	129.2 (8.1%)	128.3 (7.3%)	122.4 (2.4%)	115.6 (−3.3%)	116.1 (−2.8%)	0.003
	Low level 20 °C	124.9	122.4 (−2.0%)	118.1 (−5.5%)	123.4 (−1.2%)	123.4 (−1.2%)	117.6 (−5.8%)	0.214
	High level 4 °C	427.6	408.5 (−4.5%)	414.4 (−3.1%)	409.8 (−4.2%)	401.4 (−6.1%)	397.5 (−7.1%)	0.001
	High level 20 °C	389.5	411.0 (5.5%)	411.6 (5.7%)	404.8 (3.9%)	393.8 (1.1%)	391.3 (0.5%)	0.001

**Table 2:** Median enzyme activity (U/L) before and after three freeze–thaw cycles for PEG-Asparaginase and Erwinia asparaginase stocks. Percentage difference from baseline (%PD) is shown in parentheses. *p*-values were calculated using the Wilcoxon signed-rank test.

	Before (U/L)	After (U/L)	Percentage Difference	*p*-Values
Low-level PEG-ASNase	120.9	114.5	−5.3%	0.063
High-level PEG-ASNase	634.9	648.8	2.2%	0.156
Low-level Erwinia ASNase	105.2	103.5	−1.6%	0.787
High-level Erwinia ASNase	715.4	698.8	−2.3%	0.031

**Table 3:** Median enzyme activity (U/L) over time for PEG-Asparaginase and Erwinia asparaginase stocks in whole blood at 20 °C. Percentage difference from baseline (%PD) is shown in parentheses. *p*-values were calculated using the Friedman test.

	Baseline (U/L)	2 h (U/L)	5 h (U/L)	10 h (U/L)	24 h (U/L)	*p*-Values
Low-level PEG-ASNase	169.5	141.0 (−16.8%)	135.5 (−20.1%)	134.9 (−20.4%)	132.5 (−21.8%)	0.016
High-level PEG-ASNase	949.1	981.1 (3.4%)	922.7 (−2.8%)	930.3 (−2.0%)	960.0 (1.2%)	0.006
Low-level Erwinia ASNase	71.6	74.4 (3.9%)	75.5 (5.4%)	73.3 (2.3%)	70.5 (−1.6%)	0.009
High-level Erwinia ASNase	444.7	460.8 (3.6%)	443.5 (−0.3%)	436.4 (−1.9%)	437.0 (−1.7%)	0.033

## Data Availability

Data presented in this study is contained within the article. Further inquiries can be directed to the corresponding author.

## Abbreviations

The following abbreviations are used in this manuscript:

ALL	Acute Lymphoblastic Leukaemia
LBL	Lymphoblastic lymphoma
TDM	Therapeutic Drug Monitoring
AUH	Aarhus University Hospital
MPD	Maximal Permissible Difference
PEG	PEGylated
ASNase	Asparaginase
LL	Low level
HL	High level

## References

[1] Silverman L.B. (2001). Improved outcome for children with acute lymphoblastic leukemia: Results of Dana-Farber Consortium Protocol 91-01. Blood.

[2] Avramis V.I., Tiwari P.N. (2006). Asparaginase (native ASNase or pegylated ASNase) in the treatment of acute lymphoblastic leukemia. Int. J. Nanomed..

[3] Schore R.J., Devidas M., Bleyer A., Reaman G.H., Winick N., Loh M.L., Raetz E.A., Carroll W.L., Hunger S.P., Angiolillo A.L. (2019). Plasma asparaginase activity and asparagine depletion in acute lymphoblastic leukemia patients treated with pegaspargase on Children’s Oncology Group AALL07P4. Leuk. Lymphoma.

[4] Gottschalk Højfeldt S., Grell K., Abrahamsson J., Lund B., Vettenranta K., Jónsson Ó.G., Frandsen T.L., Wolthers B.O., Marquart H.V.H., Vaitkeviciene G. (2021). Relapse risk following truncation of pegylated asparaginase in childhood acute lymphoblastic leukemia. Blood.

[5] Gupta S., Wang C., Raetz E.A., Schore R., Salzer W.L., Larsen E.C., Maloney K.W., Mattano L.A., Carroll W.L., Winick N.J. (2020). Impact of Asparaginase Discontinuation on Outcome in Childhood Acute Lymphoblastic Leukemia: A Report From the Children’s Oncology Group. J. Clin. Oncol..

[6] European Medicines Agency (2021). Guidelines for Real-Time Therapeutic Drug Monitoring (TDM) of Asparaginase. European Union Drug Regulating Authorities Clinical Trials Database (EudraCT).

[7] European Medicines Agency A Treatment Study Protocol of the ALLTogether Consortium for Children and Young Adults (1–45 years of age) with Newly Diagnosed Acute Lymphoblastic Leukaemia (ALL) [Internet]. European Union Drug Regulating Authorities Clinical Trials Database (EudraCT). Report No.: 2018-001795–38. https://dapho.dk/wp-content/uploads/2023/05/akut-lymfoblastisk-leukaemi-alltogether.pdf.

[8] Lanvers C., Paulo Vieira Pinheiro J., Hempel G., Wuerthwein G., Boos J. (2002). Analytical validation of a microplate reader-based method for the therapeutic drug monitoring of l-asparaginase in human serum. Anal. Biochem..

[9] Fernandez C.A., Cai X., Elozory A., Liu C., Panetta J.C., Jeha S., Molinelli A.R., Relling M.V. (2013). High-throughput asparaginase activity assay in serum of children with leukemia. Int. J. Clin. Exp. Med..

[10] Yu H., Hu Q., Liu L., Xiang D., Yang G., Wang Y., Wang Z., Liu D., Liu A., Gong X. (2025). A Novel Liquid Chromatography–Tandem Mass Spectrometry Method for Monitoring Plasma l-Asparaginase Activity in Pediatric Patients With Acute Lymphoblastic Leukemia Receiving Pegaspargase Treatment. Ther. Drug Monit..

[11] (2022). Medical laboratories—Requirements for quality and competence.

[12] Kloos R.Q.H., Pieters R., Jumelet F.M.V., de Groot-Kruseman H.A., Bos C.v.D., van der Sluis I.M. (2020). Individualized Asparaginase Dosing in Childhood Acute Lymphoblastic Leukemia. J. Clin. Oncol..

